# Feasibility of Synthetic Computed Tomography Images Generated from Magnetic Resonance Imaging Scans Using Various Deep Learning Methods in the Planning of Radiation Therapy for Prostate Cancer

**DOI:** 10.3390/cancers14010040

**Published:** 2021-12-23

**Authors:** Gyu Sang Yoo, Huan Minh Luu, Heejung Kim, Won Park, Hongryull Pyo, Youngyih Han, Ju Young Park, Sung-Hong Park

**Affiliations:** 1 Department of Radiation Oncology, Samsung Medical Center, Sungkyunkwan University School of Medicine, Seoul 06351, Korea; gs.levin.yoo@samsung.com (G.S.Y.); hr.pyo@samsung.com (H.P.); youngyih.han@samsung.com (Y.H.); 2 Department of Bio and Brain Engineering, Korean Advanced Institute of Science and Technology, Daejeon 34141, Korea; luuminhhuan@kaist.ac.kr; 3 Department of Radiation Oncology, Samsung Medical Center, Seoul 06351, Korea; heejung0228@gmail.com (H.K.); smcjy.park@samsung.com (J.Y.P.)

**Keywords:** prostate neoplasm, magnetic resonance imaging, synthetic computed tomography, deep learning, radiotherapy

## Abstract

**Simple Summary:**

MRI-only simulation in radiation therapy (RT) planning has received attention because the CT scan can be omitted. For MRI-only simulation, synthetic CT (sCT) is necessary for the dose calculation. Various methodologies have been suggested for the generation of sCT and, recently, methods using the deep learning approaches are actively investigated. GAN and cycle-consistent GAN (CycGAN) have been mainly tested, however, very limited studies compared the qualities of sCTs generated from these methods or suggested other models for sCT generation. We have compared GAN, CycGAN, and, reference-guided GAN (RgGAN), a new model of deep learning method. We found that the performance in the HU conservation for soft tissue was poorest for GAN. All methods could generate sCTs feasible for VMAT planning with the trend that sCT generated from the RgGAN showed best performance in dosimetric conservation D_98%_ and D_95%_ than sCTs from other methodologies.

**Abstract:**

We aimed to evaluate and compare the qualities of synthetic computed tomography (sCT) generated by various deep-learning methods in volumetric modulated arc therapy (VMAT) planning for prostate cancer. Simulation computed tomography (CT) and T2-weighted simulation magnetic resonance image from 113 patients were used in the sCT generation by three deep-learning approaches: generative adversarial network (GAN), cycle-consistent GAN (CycGAN), and reference-guided CycGAN (RgGAN), a new model which performed further adjustment of sCTs generated by CycGAN with available paired images. VMAT plans on the original simulation CT images were recalculated on the sCTs and the dosimetric differences were evaluated. For soft tissue, a significant difference in the mean Hounsfield unites (HUs) was observed between the original CT images and only sCTs from GAN (*p* = 0.03). The mean relative dose differences for planning target volumes or organs at risk were within 2% among the sCTs from the three deep-learning approaches. The differences in dosimetric parameters for D_98%_ and D_95%_ from original CT were lowest in sCT from RgGAN. In conclusion, HU conservation for soft tissue was poorest for GAN. There was the trend that sCT generated from the RgGAN showed best performance in dosimetric conservation D_98%_ and D_95%_ than sCTs from other methodologies.

## 1. Introduction

Prostate cancer is the second most common cancer in men and the sixth leading cause of cancer-related deaths among men worldwide [1]. Radiation therapy (RT) is one of the most widely utilized local modalities along with surgery and recommended by various guidelines for the management of prostate cancer [2,3,4]. The calculation of radiation dosimetry for RT requires a map of electron densities of the anatomic sites that are converted from the Hounsfield units (HUs) provided by simulation computed tomography (CT) [5]. However, due to the unsatisfactory soft tissue contrast on CT scans, additional magnetic resonance (MR) simulations are commonly performed for accurate target delineation during RT planning for prostate cancer [6]. Although the quality of target delineation can be improved by MR imaging (MRI) fused with simulation CT, the process of co-registration of these images potentially causes systematic errors due to positional differences between CT and MRI and also due to registration uncertainty [7,8]. Moreover, additional MR scans increase the patients’ medical expenses and inconvenience as well as the workload of the medical staff. Considering these problems, RT simulation using MR alone has been proposed for prostate cancer and various methodologies have been suggested over several years [9,10,11].

Since MRI does not provide information regarding electron density, the generation of synthetic CT images (sCTs) from MRI is an essential process for MR-only simulations [9]. Various methodologies for sCT generation have been suggested and their feasibility has been evaluated in various studies over the years [10,12]. Particularly, with the recent emergence of the sCT generation technique based on the deep learning approach, MR-only simulation methods using neural networks have been developed [11,13,14,15]. Early investigations regarding sCT generation for RT simulation using deep learning techniques have focused on RT for cranial sites wherein inter-personal or temporal anatomical variations are limited. In addition, because the technique for the extraction of image features from MRI, cranial MRI has been favored due to the advances of those techniques in the brain MRI using various neural networks [16,17]. However, the use of sCTs for RT was expanded to other anatomic sites including the chest, abdomen, and pelvis [14,15,18,19]. Conventional generative adversarial networks (GANs) trained with paired image sets have been investigated most comprehensively for the generation of sCTs for RT simulations [20,21]. With the emergence of another deep learning technique using cycle-consistent GAN (CycGAN), which performs unpaired image-to-image translation, studies evaluating the feasibility of deep learning sCT models other than those based on GAN have been conducted [22,23]. However, such studies are limited to date. Herein, we evaluated the sCTs generated by various deep learning methods including GAN and CycGAN in RT for prostate cancer.

## 2. Materials and Methods

### 2.1. Patients

Simulation CT and MRI data from patients with prostate cancer who underwent RT with or without prostatectomy between January 2016 and July 2018 at Samsung Medical Center were utilized. This retrospective study was approved by the institutional review board (SMC 2018-11-087).

### 2.2. CT and MRI Acquisition

The simulation CT scans were obtained using Discovery CT590 RT (GE Healthcare, Chicago, IL, USA). The CT simulation had an 80 cm wide bore, flat couch, and a detector with 16 channels. X-rays were generated from 120 mV_p_ tube. The field of view was 65 cm. Patients were instructed to void and drink 200–300 cc of water for bladder filling at 2 h before the simulation CT scan. The CT scans were performed with helical mode and reconstructed with bone-plus filter. Contrast agent was not administrated. During the simulation CT scan, all patients were placed in the supine position and immobilized using Vac-lok. A rectal balloon with a volume of approximately 70 cc was inserted and filled with air for photon RT or with water for proton beam RT. CT scans were performed with a head-first orientation. The resolution of the CT images was 512 × 512 with a pixel spacing of 0.98 × 0.98 mm.

MR scans were performed immediately after the simulation CT scans on the same day at Samsung Medical Center. For MR simulation, Ingenia 3.0T MR simulation system (Philips Healthcare, Best, the Netherlands) with a 16-channel abdominal receiving coil and integrated posterior coil assembly was utilized. The patient setup was the same as that in the simulation CT except for the feet-first supine orientation, which allowed more flexibility than the head-first setup while positioning the anterior receiving coil. T2-weighted MRI scans were acquired in the axial plane at the resolution of a dedicated three-dimensional radiofrequency spoiled incoherent gradient 2-echo instrument (two-dimensional T2 turbo spin echo [TSE], repetition time: 6152 ms, echo time: 100 ms).

### 2.3. Network Training and sCT Generation

**Figure 1:**
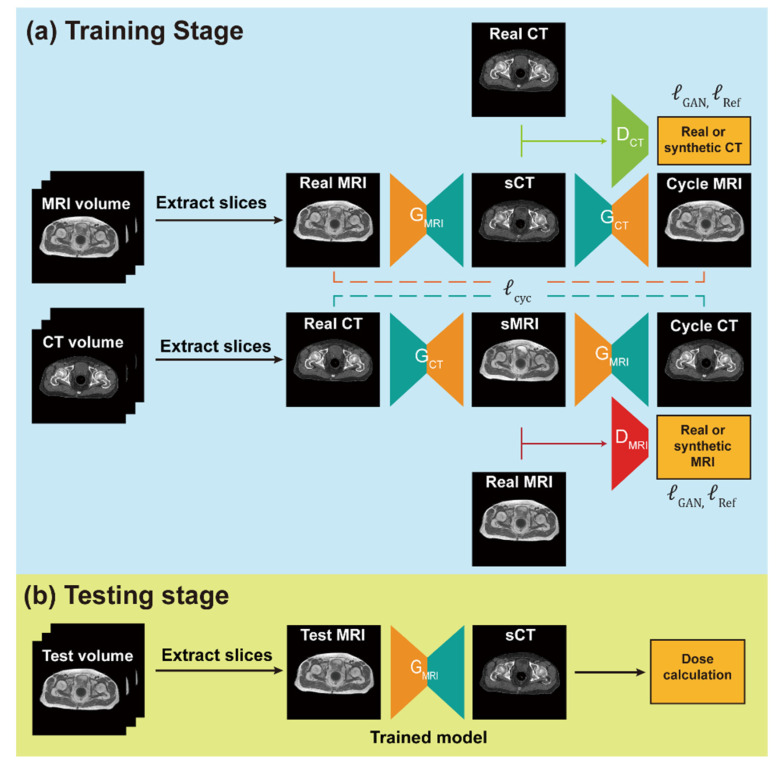

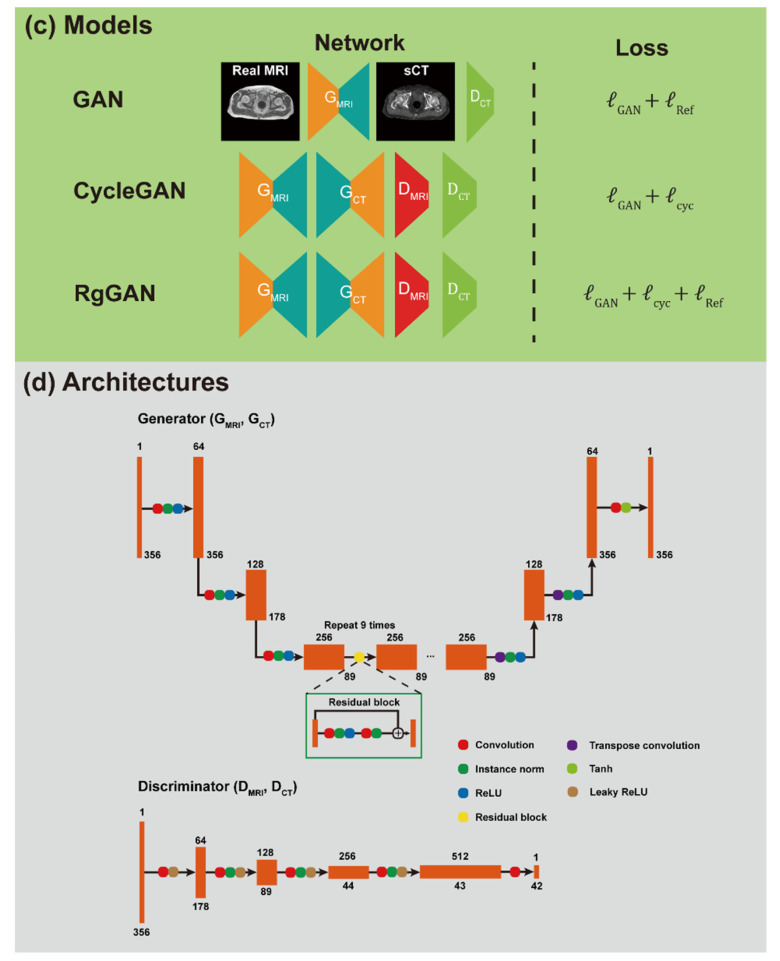
Schematic of the deep learning-based synthetic computed tomography (sCT) generation. (**a**) During training 2-dimensional (2D) axial slices are extracted from the volumes and fed to the model, which is shown as the cycle-consistent generative adversarial network (GAN) model. (**b**) The well-trained generator of magnetic resonance imaging (G_MRI_) is used to generate 2D sCT images from the test magnetic resonance imaging volume at test time. The sCT slices are reassembled to 3-dimensional volume. (**c**) The GAN model only had the G_MRI_ and discriminator (D_CT_) networks and was trained with GAN and reference loss (l_GAN_ + l_Ref_). The cycle-consistent GAN (CycGAN) model has four networks (G_MRI_, G_CT_, D_MRI_, and D_CT_) and was trained with GAN and cycle loss (l_GAN_ + l_cyc_). The reference-guided CycGAN model (RgGAN), used the same networks as the CycGAN model and was trained with GAN, cycle, and reference loss (l_GAN_ + l_cyc_ + l_Ref_). (**d**) Architectures of the generator and discriminator used in this study. The number at the top and bottom of the feature maps indicates the number of channels and spatial dimensions (width, height), respectively.

The simulation CT and MRI datasets from 93 and 20 patients were randomly selected and used as training and test sets, respectively. For the generation of sCTs, we utilized three deep learning approaches: GAN with two convoluted neural networks (CNNs) contained in one generator and one discriminator, CycGAN with four CNNs in two generators and two discriminators, and reference-guided CycGAN (RgGAN), which performed further adjustment of the sCTs generated by CycGAN with available paired images. The schematic flow for sCT generation based on the deep learning methods is shown in Figure 1.

### 2.4. Data Pre-Processing

Initially, the data were manually inspected to remove those with unusual artifacts, resulting in the selection of 113 pairs of MRI/CT volumes among 119 pairs. To process the data for training the networks, we resampled both MRI and CT volumes to a common 1.3 × 1.3 × 2.5 mm^3^ spacing and registered MRI to CT with affine transformation using the implementation in the SimpleElastix library. MR slices that missed more than 5% of the body information due to the registration process were discarded. We cropped the central 384 × 384 pixels in the axial plane to remove the background. Simple thresholding and three-dimensional connected component analysis was applied to the resampled CT volumes to extract only the body and remove irrelevant features such as couch and background intensity. The entire range of CT intensities [−1024, 3071] was then normalized to [−1, 1]. A similar processing method was utilized for the MRI data with the exception of intensity normalization. To normalize the MRI data, we subtracted the mean divided by 2.5 times the standard deviation of the voxels inside the body (98.7 percentile) and clipped the values to [−1, 1]. The processed data were split randomly into 86, 7, and 20 subjects for training, validation, and testing, respectively. For MRI, 6467 training images, 538 validation images, and 1582 test images were used. For CT, 6983 training images, 538 validation images, and 1582 test images were used.

### 2.5. Network Training and sCT Generation

We used the processed data to train and compare three different deep learning models for sCT generation. The first model involved a GAN-based method with a single generator and a single discriminator [24]. This model was trained with a combination of the mean absolute error (MAE) between the sCTs and reference CT images and the adversarial loss with a ratio of 15:1. The adversarial loss is based on the least-square GAN loss and is defined as:

(1) $${ℒ}_{\mathrm{adv},\mathrm{dis}}=E_{x\in \mathrm{CT}}{\left(D_{CT}\left(x\right)-1\right)}^{2}+E_{y\in \mathrm{MRI}}{\left(D_{CT}\left(G_{MRI}\left(y\right)\right)\right)}^{2}$$

(2) $${ℒ}_{\mathrm{adv},\mathrm{gen}}=E_{y\in \mathrm{MRI}}{\left(D_{CT}\left(G_{MRI}\left(y\right)\right)-1\right)}^{2}$$

for the discriminator and generator, respectively.

The second model was a CycGAN-based [25] method with two generators and two discriminators. The loss of cycle-consistency enables unpaired training, which was applicable in our case due to the imperfect registration between MR and CT. Hence, this model was trained without the MAE loss, using only the standard CycGAN losses, namely the cycle-consistency loss and the adversarial loss with a ratio of 10:1. The adversarial loss for the CycGAN included the terms for distinguishing real and fake MRI images:

(3) $$\begin{array}{c} {ℒ}_{\mathrm{adv},\mathrm{dis}}=E_{x\in \mathrm{CT}}{\left(D_{CT}\left(x\right)-1\right)}^{2}+E_{y\in \mathrm{MRI}}{\left(D_{CT}\left(G_{MRI}\left(y\right)\right)\right)}^{2}\\ +E_{y\in \mathrm{MRI}}{\left(D_{MRI}\left(y\right)-1\right)}^{2}+E_{x\in \mathrm{CT}}{\left(D_{MRI}\left(G_{CT}\left(x\right)\right)\right)}^{2} \end{array}$$

(4) $${ℒ}_{\mathrm{adv},\mathrm{gen}}=E_{x\in \mathrm{CT}}{\left(D_{MRI}\left(G_{CT}\left(x\right)\right)-1\right)}^{2}+E_{y\in \mathrm{MRI}}{\left(D_{CT}\left(G_{MRI}\left(y\right)\right)-1\right)}^{2}$$

The third model, referred to as RgGAN in this study, followed the CycGAN formulation with the addition of MAE for available paired images during training with a weight of 100. The additional use of MAE for available paired images in CycGAN was hypothesized to improve the performance of the CycGAN model by enhancing the similarity between the sCTs and the original CT images. The Adam optimizer was used to train all networks with momentum parameters of β1 = 0.5 and β2 = 0.999 on a graphic processing unit (GeForce GTX 1080Ti, NVIDIA Corp, Santa Clara, CA, USA). The batch size was 2 and the learning rate was 0.0002 for the first 10 epochs and linearly decayed to 0 for the next 30 epochs. During training, the images were randomly cropped to 356 × 356 before being processed by the network. During testing, the images were centrally cropped to the same size, processed by the network, padded with 0 to 384 × 384, and resampled to the CT spacing to be consistent with the preprocessing that was used during training. The schematic flow for the training and testing of sCT generation with a CycGAN-based network and the network architecture are shown in Figure 1. Each generator had 11.3 million parameters and each discriminator had 2.7 million parameters.

### 2.6. Assessment of sCT Quality

HUs of the sCTs generated from the three networks were compared with those of the original simulation CT images by calculating the mean error (ME) and the MAE. Dice similarity coefficients (DSCs) and Hausdorff distances for body, bony structure, rectal balloon, and soft tissue, segmented in each original CT and sCT image were calculated to compare the structural metrics between the sCTs and the original simulation CT images according to the networks [26]. The peak signal-to-noise ratio (PSNR) was also computed to measure the reconstruction quality.

For dosimetric evaluation, original CT-based RT dose distributions were recalculated on the sCTs generated from the three networks. The clinical target volumes (CTVs) were delineated as prostate with or without seminal vesicle according to the tumor extent for definitive RT or prostate bed for RT after radical prostatectomy on the original simulation CT images. The CTVs were expanded by 0.3–0.5 mm to generate the planning target volume (PTV). The organs at risk (OARs) including the bilateral femoral heads, bladder, penile bulb, and rectum were delineated. In addition, body contours, bony structures and soft tissues which was generated by extraction of bony structures from body contours were also delineated via the auto-contouring tool using the threshold of HU units with manual adjustment on original simulation CT and all of the sCTs for the assessment of the sCT quality according to the anatomic subsites. Volumetric modulated arc therapy (VMAT) was selected as the techniques for RT. Pinnacle version 9.10 (Philips Radiation Oncology Systems, Philips Healthcare, Best, the Netherlands) was used for target volume delineation and dosimetric calculation. The dose differences between the original simulation CT images and sCTs were evaluated by comparing the dose-volume histogram (DVH) metrics including D_98%_, D_95%_, D_50%_, D_5%_, and D_2%_ of the CTV, PTV, and OAR between the original simulation CT images and the sCTs. Three-dimensional global gamma pass rates were computed using the original simulation CT dose as a reference and a dose threshold of 10%. The gamma pass rates were evaluated with 2 mm and 3 mm distances to agreement, 2% dose difference and 2 mm distance to agreement, and 2% dose difference.

### 2.7. Statistical Analysis

The chi-squared test or Fisher’s exact test was used to compare the patients’ characteristics between the training/validation and testing groups. One-way analysis of variance with Bonferroni correction was performed to compare continuous variables among the sCTs or the original simulation CT images. Statistical significance was set at a two-sided *p*-value < 0.05. Statistical analyses were performed using the IBM SPSS Statistics version 25 (IBM Corp., Armonk, NY, USA).

## 3. Results

### 3.1. Patients’ Characteristics

The characteristics of the training, validation group and testing group are summarized in Table 1. No statistically significant differences were observed in median age, stage, prostatectomy status, or RT modalities between the groups.

### 3.2. Image Quality

**Figure 2:**
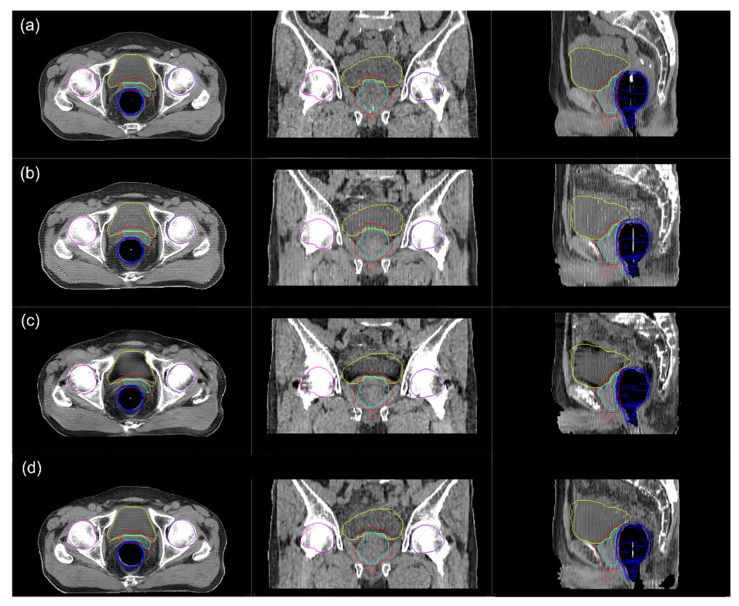
Example of original (**a**) and synthetic computed tomography generated from generative and adversarial network (GAN) (**b**), cyclo-consistent GAN (**c**) and reference-guided cyclo-consistent GAN (**d**) (left; axial view, middle; coronal view; right sagittal view).

**Figure 3:**
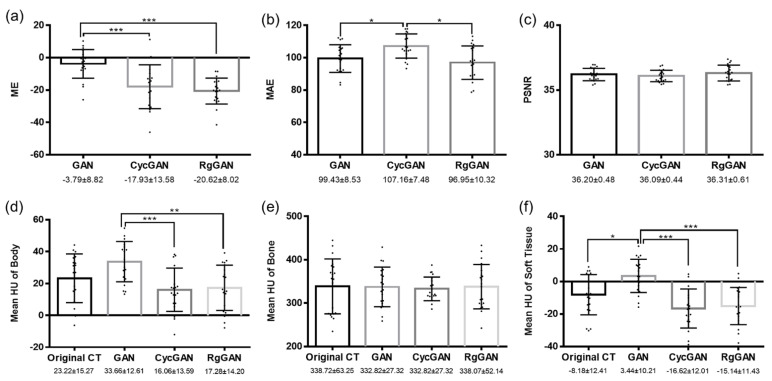
Comparison of mean error (**a**), mean absolute error (**b**), and peak signal-to-noise ratio (**c**) and mean Hounsfield units of original computed tomography and synthetic computed tomography for body (**d**), bone (**e**), and soft tissue (**f**) according to the deep learning techniques. Abbreviations: GAN, generative adversarial network; CycGAN, cyclo-consistent generative adversarial network; RgGAN, Reference-guided cyclo-consisten generative adversarial network; ME, mean error; MAE, mean absolute error; PSNR, peak signal-to-noise ratio. * 0.01 < *p* < 0.05, ** 0.001 < *p* < 0.01, *** *p* < 0.001.

**Figure 4:**
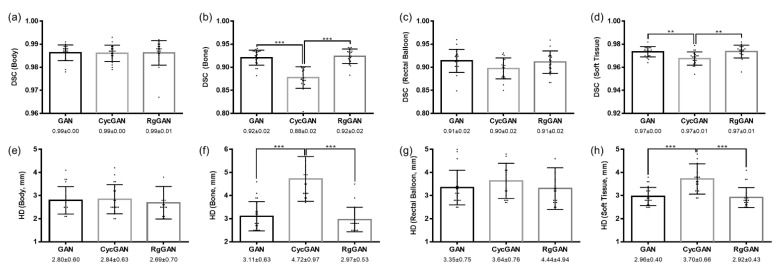
Comparison of dice-similarity coefficients for body (**a**), bone (**b**), rectal balloon (**c**), and soft tissue (**d**) and Hausdorff’s distance (HD) for body (**e**), bone (**f**), rectal balloon (**g**), and soft tissue (**h**) in synthetic computed tomography according to the deep learning techniques. Abbreviations: DSC, dice-similarity coefficient; GAN, generative adversarial network; CycGAN, cyclo-consistent generative adversarial network; RgGAN, Reference-guided cyclo-consistent generative adversarial network; HD, Hausdorff distance. * 0.01 < *p* < 0.05, ** 0.001 < *p* < 0.01, *** *p* < 0.001.

Figure 2 shows an example of original simulation CT and sCTs generated based on GAN, CycGAN, and RgGAN. Comparisons of ME, MAE, and PSNR among the sCTs are shown in Figure 3. While there were significant differences in mean ME between the sCT from GAN and that from CycGAN (*p* < 0.001) or RgGAN (*p* < 0.001), the mean MAE was significantly higher in the sCTs from CycGAN than in the sCTs from GAN (*p* = 0.023) and RgGAN (*p* = 0.002). No significant differences were observed in PSNR among the sCTs. The mean HU values of various anatomic subsites according to the original CT images or sCTs are compared in Figure 3. For soft tissue, a significant difference in the mean HUs was observed only between the original CT images and the sCTs from GAN (*p* = 0.03). For other sites, no significant difference was observed in the mean HUs between the original CT images and the sCTs. Figure 4 shows the comparison of DSCs among the sCTs according to the subsites. For the bone and soft tissues, the mean DSCs of CycGAN were significantly lower than those of GAN (*p* < 0.001 for bone and *p* = 0.003 for soft tissue) and RgGAN (*p* < 0.001 for both bone and soft tissue). Similarly, the Hausdorff distances were significantly higher in CycGAN for bone and soft tissue than in GAN (*p* < 0.001 for both bone and soft tissue) and RgGAN (*p* < 0.001 for both bone and soft tissue) as shown in Figure 4.

### 3.3. Dosimetric Comparison

**Figure 5:**
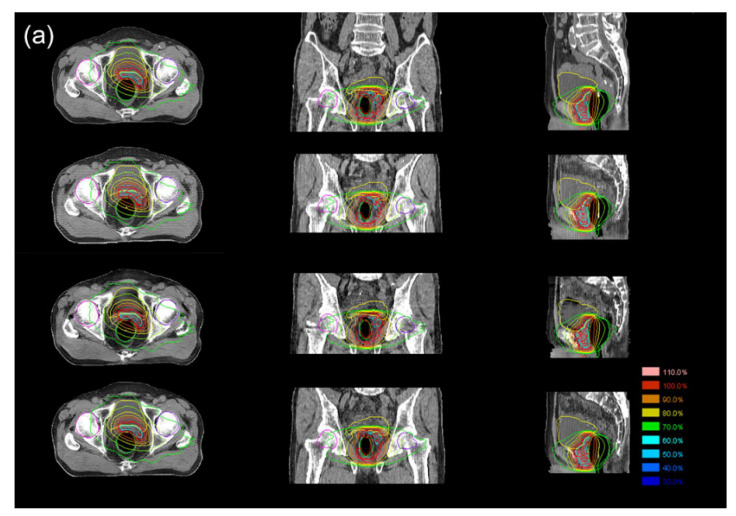

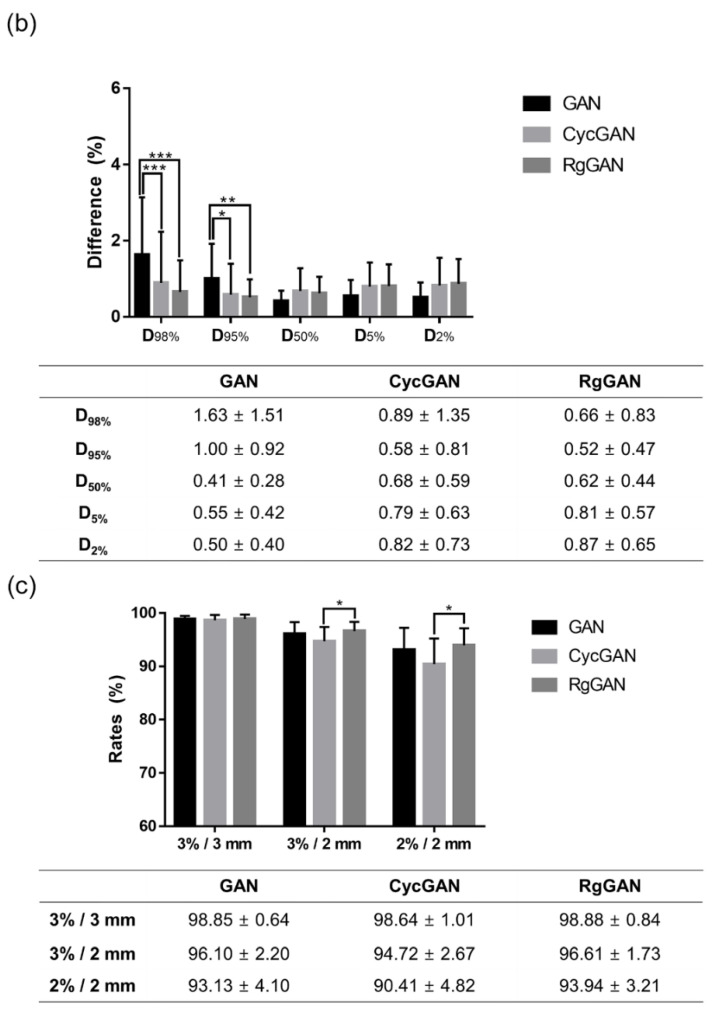
(**a**) Example for the comparison of dosimetric distributions on original and synthetic computed tomography according to the deep learning techniques. Dosimetries of axial, coronal, and sagittal views for volumetric modulated arc therapy (VMAT) on original computed tomography (CT), and synthetic CTs (sCT) based on generative adversarial network (GAN), cyclo-consistent GAN (CycGAN), and Reference-guided CycGAN (RgGAN). The dosimetric distributions are represented on axial (right), coronal (middle), and sagittal (right) slices of original CT, and sCTs generated from GAN, CycGAN, RgGAN from the uppermost row to lowermost row (**a**). (**b**) Difference of dosimetric parameters in planning target volume on sCTs according to the deep learning techniques comparing with original plan for VMAT. (**c**) Comparisons of gamma-pass rates among the sCTs according to the deep learning techniques for VMAT. Abbreviations: PTV, planning target volume; VMAT, volumetric modulated arc therapy; PBT, proton beam therapy; GAN, generative adversarial network; CycGAN, cyclo-consistent generative adversarial network; RgGAN, Reference-guided cyclo-consistent generative adversarial network. * 0.01 < *p* < 0.05, ** 0.001 < *p* < 0.01, *** *p* < 0.001.

Figure 5 shows the dosimetric distributions in an example case, the differences in DVH parameters for PTV between original CT and sCT, and the gamma-pass rates. The mean differences in various DVH parameters for PTV were less than 2% in the original CT. No significant differences were observed among the sCTs except for differences in D_98%_ and D_95%_ between GAN and other deep learning methods (Figure 5). The gamma-pass rates were consistently lowest in the sCTs from CycGAN, with a significant difference compared to the sCTs from RgGAN for gamma indices of 3%/2 mm and 2%/2 mm. For OARs including the rectum, bladder, bilateral femurs, and penile bulb, the DVH parameters were not significantly different among the sCTs (Figure S1).

## 4. Discussion

Previous investigations regarding feasibility of the sCTs generated from MRI using deep learning technologies for MR-only RT simulations have mainly focused on the sCTs generated by GAN, which requires paired image sets in the training process [24]. However, simulation CTs and planning MRIs in the training set were not perfectly matched pairs. Therefore, another deep learning methodology, CycGAN, has recently been evaluated for the generation of sCTs for RT simulation. It does not require paired image sets in the training [24]. In the present study, we evaluated the feasibility of CycGAN and GAN for the generation of sCTs in an MR-only RT simulation for prostate cancer in VMAT planning. Furthermore, a third model, referred to as RgGAN, was also evaluated in our study. It uses the CycGAN formulation, but also utilizes the MAE between available paired reference images. In the present study, sCTs based on RgGAN showed the tendency to improve the quality of those from CycGAN in terms of the conservations in the radiological parameters of anatomic subsite, especially soft tissue and conserve DVH parameters regarding higher dose comparing with those from GAN or CycGAN.

In the evaluation of sCT quality, CycGAN showed a significantly higher MAE and lower ME when compared with GAN. In addition, significantly lower DSCs and higher Hausdorff distances of bone and soft tissue were observed in CycGAN when compared with GAN. These significant differences in MAE, DSCs, and Hausdorff distances were maintained between GAN and RgGAN. These results suggest that GAN performed better in sCT generation than CycGAN and the limitations of CycGAN were complemented using paired reference images in addition to the formulation of CycGAN in RgGAN. However, these parameters are indicators for the similarities between original CT images and sCTs originating from the corresponding MRIs, which have intrinsically different geometries due to inter-scan variations between the original CT and MR images and intra-scan variations during the MRI scan. While training using GAN minimizes the loss of sCTs from the paired original CT images, CycGAN does not specifically concern the loss from its paired original CT image. Therefore, these parameters could potentially indicate higher similarities between GAN-generated sCTs and the original CT images when compared with those between CycGAN-generated sCTs and the original CT images. Previous studies comparing the sCTs generated from GAN and CycGAN showed a similar tendency wherein MAE tended to be higher in the sCTs from CycGAN than in those from GAN. These studies also suggested that inter-scan geometric distortion between CT and MRI scans and intra-scan deformities during MRI could result in a worse performance of CycGAN when compared with GAN [13,27]. Contrary to the results regarding MAE, DSC, and Hausdorff distances, the difference in the mean HUs of soft tissues was statistically significant only between the original CT images and the sCTs generated from GAN. The comparison of mean HUs between original CT images and corresponding sCTs according to the anatomical subsites can exclude the geometric distortion in the analyses and evaluate the quality in terms of HU conservation according to the anatomical subsites. Therefore, these results imply that the performance in terms of conservation of HU distribution in the soft tissue could be potentially superior in the sCTs from CycGAN or RgGAN when compared with those from GAN.

Despite the differences in quality among the sCTs, most of the calculated dosimetric parameters such as PTVs or OARs were not significantly different among the sCTs from the three deep learning approaches. The gamma indices also indicated acceptable conservation of dosimetric distribution. Since the RT plans that were recalculated on the sCTs were optimized for the target volume on the original CT images showing geometrical distortion compared to the sCTs, the differences in the sCT dosimetric parameters could also be affected by the geometrical distortions compared to the original CT images. Therefore, it could be predicted that the dosimetry conservation would be best in the sCT from GAN or RgGAN which followed the paired original CT more than CycGAN. However, the differences in the DVH parameters of PTV for higher doses (D_98%_ and D_95%_) were significantly higher in the plans based on sCTs from GAN rather than in those based on sCTs form CycGAN or RgGAN when compared with the plans on the original CT images. Between the CycGAN and RgGAN, RgGAN showed higher conservation in the DVH parameters of PTV for higher does although there was no statistical significance. These results suggest that a lower amount of geometrical similarity between the sCTs and the original CT images does not necessarily lead to inferior dosimetric conservation on the sCTs for VMAT planning. Instead, the lower conservation of HU in anatomical subsites could lead the lower conservation of dosimetric distribution as our result showing that the difference in mean HU and DVH parameters for higher does for PTV. Further studies would be necessary to confirm the hypothesis.

In terms of parameters such as ME, MEA, or gamma indices, our results do not seem to show improved performance when compared with the previous literature [15,18,19]. Direct comparisons among relevant studies are difficult, since sCT generation is dependent on the protocols of the dataset acquisition for training, which are extremely diverse among different studies. Particularly, spatial blurring can occur in MRI depending on the sequence. In the present study, T2-TSE MRI sequence was used in the training, which can result in an image blurring effect due to T2-related signal loss on late echoes [28,29]. Since the quality of sCTs depends on that of the original MRI, adequate MRI sequences for sCT generation need to be established. Alternatively, it is worth considering the use of a combination of multiple MRI sequences that complement sCT generation [15,16].

The present study has some limitations. It involved a small number of datasets for the training, potentially leading to suboptimal sCT generation performance. The results are from a single institution and further external validation studies are required to evaluate whether the protocol in our study can be generalized. In addition, since the delineations of anatomic subsites were involved by manual adjustments, there could be biases in the comparisons of the sCTs in terms of the mean HU, DSC, or Hausdorff distances according to the anatomic subsites. Finally, since the evaluated plans were limited only to the prostate or the prostate bed, the feasibility of sCTs in RT planning of larger target volumes remains to be investigated.

## 5. Conclusions

CycGAN and RgGAN may improve the similarity of sCTs with the original CT images in terms of soft tissue HU distribution when compared with GAN. GAN, CycGAN, and RgGAN could generate sCTs from T2-weighted MR images that are feasible for use VMAT planning by achieving clinically acceptable dosimetric conservation when compared with the original RT plans. There was a trend that sCT generated from the RgGAN showed best performance in terms of the HU accuracy in soft tissue and dosimetric conservation D_98%_ and D_95%_ than sCTs from other methodologies.

## Figures and Tables

**Table 1:** Patients’ characteristics of training and test sets (*N* = 113).

Characteristics	Number (%)		*p* Value
	Training/Validation Group (*n* = 93)	Test Group (*n* = 20)	
Median age (range)	72 (58–82)	69 (58–88)	0.908
T stage			0.476
1–2	32 (34.4)	9 (45.0)	
3–4	61 (65.6)	11 (55.0)	
N stage			0.778
0	68 (73.1)	16 (80.0)	
1	25 (26.9)	4 (20.0)	
M stage			1.000
0	85 (91.4)	19 (95.0)	
1	8 (8.6)	1 (5.0)	
Prostatectomy			0.443
Yes	9 (9.7)	3 (15.0)	
No	84 (90.3)	17 (85.0)	
Radiotherapy modality			0.379
X-ray	86 (92.5)	17 (85.0)	
Proton	7 (7.5)	3 (15.0)	

## Data Availability

The data presented in this study are available on request from the corresponding author.

## Supplementary Materials

The following are available online at https://www.mdpi.com/article/10.3390/cancers14010040/s1. Figure S1: Difference of dosimetric parameters in organs at risk on synthetic computed tomography according to the deep learning techniques comparing with original plan for volumetric modulated arc therapy and proton beam therapy.
