# Supplementary material for: Feasibility of Synthetic Computed Tomography Images Generated from Magnetic Resonance Imaging Scans Using Various Deep Learning Methods in the Planning of Radiation Therapy for Prostate Cancer
Source: Cancers (Basel). 2021 Dec 23;14(1):40. doi: 10.3390/cancers14010040 (PMC8750723; doi:10.3390/cancers14010040)
Supplement: Supplementary file 1 [file cancers-14-00040-s001.zip › cancers-1499097-supplementary.pdf]

cancers

MDPI

Article

Feasibility of Synthetic Computed Tomography Images Generated from Magnetic Resonance Imaging Scans Using Various Deep Learning Methods in The Planning of Radiation Therapy for Prostate Cancer

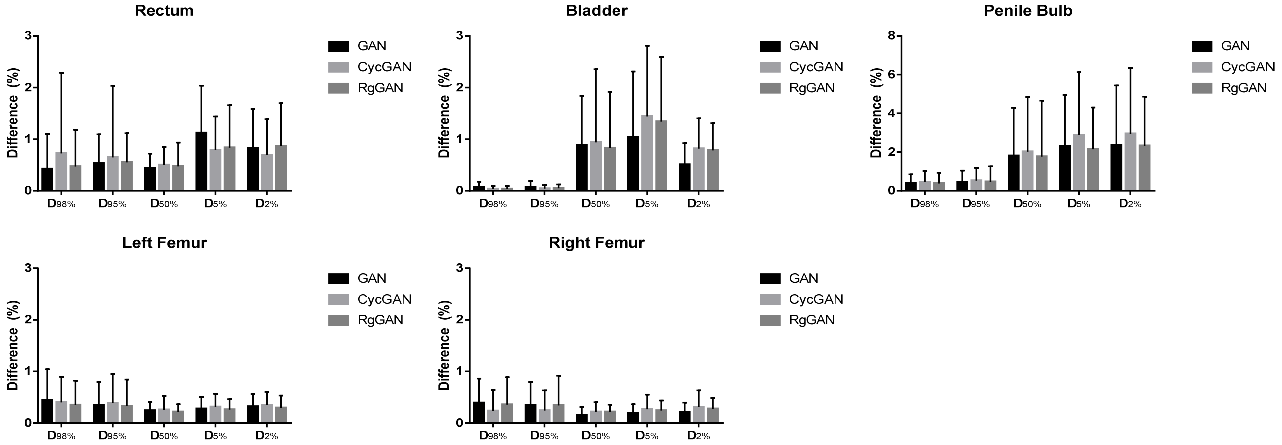

Figure S1. Difference of dosimetric parameters in organs at risk on synthetic computed tomography according to the deep learning techniques comparing with original plan for volumetric modulated arc therapy and proton beam therapy.

Abbreviations: GAN, generative adversarial network; CycGAN, cyclo-consistent generative adversarial network; RgGAN, Reference-guided cyclo-consistent generative adversarial network
